# Prognostic significance of CIP2A expression in solid tumors: A meta-analysis

**DOI:** 10.1371/journal.pone.0199675

**Published:** 2018-07-25

**Authors:** Min Tang, Jiao-Feng Shen, Ping Li, Li-Na Zhou, Ping Zeng, Xi-Xi Cui, Min-Bin Chen, Ye Tian

**Affiliations:** 1 Department of Radiotherapy and Oncology, the Second Affiliated Hospital of Soochow University, Institute of Radiotherapy & Oncology, Soochow University, Suzhou, Jiangsu, China; 2 Department of Radiotherapy and Oncology, Kunshan First People’s Hospital Affiliated to Jiangsu University, Kunshan, Jiangsu Province, China; 3 Department of Oncology, the Second Affiliated Hospital of Soochow University, Suzhou, Jiangsu, China; Sapporo Ika Daigaku, JAPAN

## Abstract

CIP2A, cancerous inhibitor of protein phosphatase 2A, was initially recognized as an oncoprotein. Recently several studies revealed that CIP2A could function as a prognosis biomarker, however, the result remained not comprehensive, partly due to small number of patients included individually. Here we carried out a meta-analysis of published studies to assess the prognostic significance of CIP2A in solid tumors. All eligible studies were identified through searching PubMed, Embase and Web of Science database. In this meta-analysis, 22 studies involving 4,579 participants were included, and we verified that CIP2A over-expression was significantly related with poor overall survival (pooled HR = 1.844, 95% CI = 1.528–2.225, P<0.001) and short disease free survival (pooled HR = 1.808, 95% CI = 1.591–2.055, P<0.001) in solid tumors. Additionally, subgroup analysis suggested that the trend of a poor overall survival with an increased CIP2A expression was present in East-Asian and European patients, as well as in lung cancer and colorectal cancer. To sum up, CIP2A over-expression was associated with poor survival in human solid tumors and might be a predictive factor of poor prognosis.

## Introduction

Reversible phosphorylation was well known to regulate protein function and transfer cell signal. Protein kinases and phosphatases were two classes of enzymes that control protein phosphorylation status lead to a variety of human diseases including cancer [1]. Serine/threonine protein phosphatase 2A (PP2A) was a cancer inhibitor which play a critical role in inhibition of cell malignant transformation via negatively regulating several major signaling pathways involved in the cancer progression [2]. Also, reduced PP2A activity can be considered as the switch in signal transduction and mediator of drug resistance and immune surveillance [3]. PP2A is a heterotrimeric complex comprised of a scaffolding subunit A, a regulatory subunit B, and a catalytic subunit C. Several strategies in restoration of this phosphatase function might be applied to targeted therapies, such as affecting its post translational modifications and its endogenous inhibitors [4]. As one of the most important endogenous inhibitor of PP2A, cancerous inhibitor of protein phosphatase 2A (CIP2A) was identified as human oncoprotein which was encoded by the KIAA1524 gene [5]. CIP2A interacted directly with the oncogenic transcription factor MYC, inhibited PP2A activity toward MYC serine 62 (S62), and thereby prevented MYC proteolytic degradation [6]. This function contributed to consequent effects on different signaling pathway such as PI3K-AKT-mTOR pathway and RAS-MEK-ERK pathway [7]. In addition, existing evidences have shown that CIP2A conductively increase cell proliferation and the growth of xenografted tumors in various cancers [6, 8, 9]. High CIP2A expression is seen in over 70% tumor patient specimen and its overexpression can predict response and resistance to chemotherapeutics [10].

An increased expression of CIP2A had been detected in multiple malignancies, such as gastric cancer[11, 12], breast cancer[13, 14], prostate cancer[15, 16], lung cancer[17], papillary thyroid carcinoma[18] and head and neck squamous cell carcinomas [19]. Clinical relevance of CIP2A high-expression would be further considered as a prognostic marker in cancer patients. Certain studies have proved CIP2A expression in tumor cells was correlated with poor prognosis [9, 12–14, 20–36]. However, some have shown no association with patient prognostic significance [37, 38] and even improved prognosis in nodular melanomas [29]. The results of those individual studies were controversial. Hence, we performed this comprehensive meta-analysis to evaluate the prognostic value of CIP2A in solid tumors.

## Materials and methods

### Publication search

This meta-analysis was conducted under the Preferred Reporting Items for Systematic Reviews and Meta-Analyses (PRISMA) guidelines[39]. PubMed, Embase, and Web of Science databases were searched (up to September 15, 2017) using the search terms: (KIAA1524 protein OR “Cancerous inhibitor of protein phosphatase 2A” OR CIP2A) AND (neoplasms OR neoplasms OR cancer OR tumor OR carcinoma) AND (prognosis OR mortality OR survival OR Survival OR predict OR outcome). All potentially eligible studies were retrieved and their bibliographies were carefully scanned to identify other eligible studies. Extra studies were identified by a hand search of the references cited in the original studies. If there were multiple studies based on the same patient population, we included the published report with the largest sample size or the most recent one. Only studies published in English were included in this meta-analysis.

### Inclusion and exclusion criteria

As proposed by the PRISMA, we used the participants, interventions, comparators, outcomes, time frames for follow-up, settings in which the interventions are delivered, and study designs to specify the eligibility criteria. Eligible studies had to meet the following inclusion criteria: (a) prospective or retrospective comparative cohort studies which evaluating CIP2A expression for predicting prognosis in human cancer, (b) sufficient information to estimate individual hazard ratios (HRs) with 95% confidence intervals (CIs) or enable calculation of these statistics from the data presented, (c) classify CIP2A expression as “high” and “low” or “positive” and “negative”. The exclusion criteria included the following: (a) letters, review articles, case reports, conference abstracts, experimental studies, editorials and expert opinions, (b) insufficient survival data for further quantitative analysis, (c) non-human research, (d) the follow-up duration was shorter than 3 years.

### Data extraction and quality assessment

Two primary investigators independently evaluated and extracted data from each study using a standardized form. All studies were double-checked and disagreements were discussed and resolved by consensus in a meeting with a third investigator. The meta-analysis of CIP2A expression was based on two outcome endpoints: OS and DFS. The following information was recorded from the including studies: author, publication year, country of origin, number of patients, types of cancer, tumor stage, detection method, cut-off value for CIP2A positivity, OS and DFS. If data from any of the earlier categories were not reported in the primary study, items were reported as “not available”. The multivariate HR was extracted to assess prognostic value of CIP2A expression. Digitizer V4.1 was then used to obtain survival data when only Kaplan-Meier curves were presented in the studies, and applying Tierney’s method to estimate the HRs and 95%CIs[40]. All studies were assessed by Newcastle-Ottawa Scale (NOS) and studies with 6 scores or more were considered as high methodological quality[41].

### Statistical analysis

Pooled HRs and 95% CIs for their outcome endpoints (OS and DFS) from each eligible study were used to evaluate the relevance between CIP2A expression and prognosis. The heterogeneity of pooled results was analyzed by using Cochran’s Q-test and Higgins’s I^2^ statistics[42]. P value >0.10 and I^2^ <50% suggested a lack of heterogeneity among studies. When no significant heterogeneity existed among studies, the fixed-effects applied. Otherwise, the random effects model was selected[43]. Subgroup analysis was performed on the basis of case population and cancer type. Funnel plots and the Egger’s test were conducted to estimate the potential publication bias[44]. If a publication bias did exist, its influence on the overall effect was assessed by the Duval and Tweedie’s trim and fill method[45]. We performed sensitivity analysis to estimate if certain individual article could influence the overall result. All statistical analyses were carried out with Stata 14.0 (Stata Corporation, College Station, Texas, USA).

## Results

### Demographic characteristics

**Fig 1:**
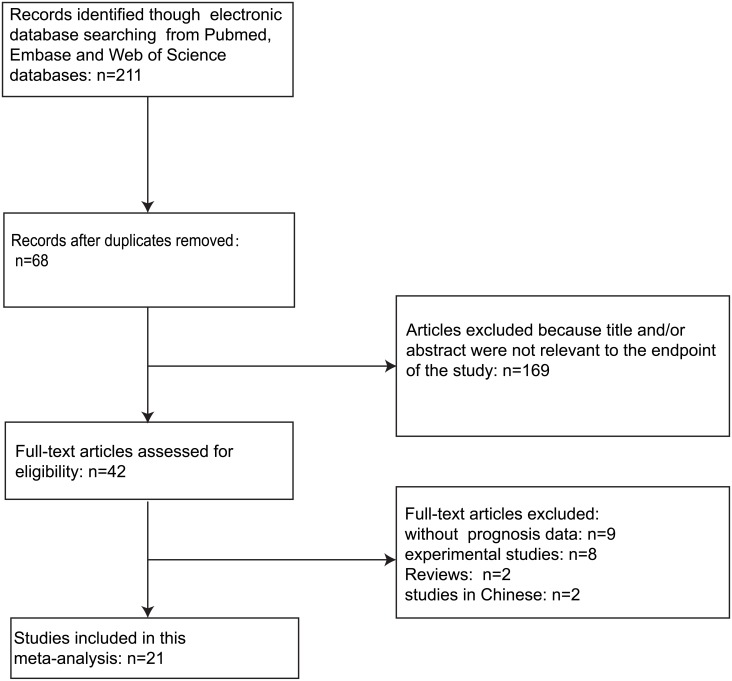
The flow chart of the selection process in our meta-analysis.

A total of 212 articles were retrieved by a literature search of the PubMed, Embase, and Web of Science databases, using the search strategy as follow: (KIAA1524 protein OR “Cancerous inhibitor of protein phosphatase 2A” OR CIP2A) AND (neoplasms OR neoplasms OR cancer OR tumor OR carcinoma) AND (prognosis OR mortality OR survival OR Survival OR predict OR outcome). The flow diagram of literature retrieval procedure is shown in Fig 1. On account of repeated data, 68 records were removed. After browsing the retrieved titles and abstracts, 101 records were excluded due to no relevant endpoint provided. After a full-text review of the remaining articles, 22 studies met the inclusion norm. The main features of these eligible studies were summarized in Table 1. In total, the 22 studies provided a sample of 4579 patients to assess the relationship between CIP2A expression and solid tumor prognosis. The median sample-size was 101, with a wide range from 37 to 1280. Among all cohorts, China (n = 16) was the major source region, followed by Finland (n = 3), Germany (n = 1), Spain (n = 1) and Norway (n = 1). As for the cancer type, four studies evaluated lung cancer, four studies evaluated colorectal cancer, two studies evaluated renal cell carcinoma, two studies evaluated hepatocellular carcinoma, two studies evaluated breast cancer, one study evaluated gastric cancer, one study evaluated tongue cancer, one study evaluated bladder cancer, one study evaluated nasal pharyngeal cancer, one study evaluated pancreatic ductal adenocarcinoma, one study evaluated melanoma, one study evaluated ovarian cancer, and one study evaluated cholangiocarcinoma. Overall, 18 studies focused on overall survival (OS), one study focused on disease free survival (DFS) and the rest three focused on both OS and DFS.

**Table 1:** Characteristics of studies included in the meta-analysis.

First author	Year	Country	case	Cancer type	Disease Stage	Detection	CIP2A positive(%)	Provided information on cutoff value	outcome endpoints	NOS score
Dong ZQ[22]	2011	China	90	NSCLC	I-IV	IHC	72.20	score > 0(range of 0–12)	OS	6
Ren J[20]	2011	China	85	Renal cell carcinoma	I-IV	IHC	70.00	low (0–1) or high (2–3)	OS	7
C Bockelman[24]	2011	Finland	73	tongue cancer	T1-2N0M0	IHC	84.50	low (0–2) or high (3)	OS	7
C Bockelman[33]	2011	Finland	524	serous ovarian cancer	I-IV	IHC	58.60	score >1(range of 0–3)	OS	7
He H[25]	2012	China	136	HCC	I-IV	IHC	70.30	low (0–4) or high (5–12)	OS, DFS	6
C Bockelman[37]	2012	Finland	540	colorectal cancer	I-IV	IHC	87.90	score >1(range of 0–3)	OS	8
Huang PZ[34]	2012	China	136	HCC	I-IV	RT-PCR	77.90	NA	OS, DFS	6
Teng HW[23]	2012	Taiwan	167	Colon Cancer	I-IV	IHC	68.30	H score ≥ 150(range of 0–300)	OS	7
Xu P[21]	2012	China	97	NSCLC	I-IV	IHC	76.29	score > 0(range of 0–12)	OS	7
Xu P[36]	2013	China	57	cholangiocarcinoma	I-IV	IHC	78.95	score > 0(range of 0–12)	OS	6
Sung WW[30]	2013	Taiwan	98	lung adenocarcinoma	I-III	RT-PCR	50.00	NA	OS	7
Wiegering A[27]	2013	Germany	104	Colorectal cancer	I-IV	RT-PCR	NA	above median fold expression value of 10,5 above normal tissue	OS	8
Wang L[26]	2013	China	96	pancreatic ductal adenocarcinoma	I-IV	IHC	56.30	the percentage of mild staining cells was greater than >10% of tumor cells	OS	6
Xue YJ[99]	2013	China	117	bladder urothelial cell carcinoma	Ta,T1-4	IHC	72.60	score > 0(range of 0–12)	OS	6
Yu GZ[13]	2013	China	1280	breast cancer	I-IV	IHC	77.60	≥7. of morphologically unequivocal neoplastic cells discretely expressed CIP2A in their cell cytoplasmic	DFS	7
Liu N[28]	2014	China	280	NPC	I-IV	IHC	65.70	score > 2(range of 0–4)	OS, DFS	7
Liu Z[32]	2014	China	57	lung cancer	I-IV	IHC	63.79	NA	OS	6
Flørenes VA(a)[29]#	2015	Norway	51	nodular melanoma	NA	IHC	67.00	score >2 in cytoplasm and >0 in nucleus(range of 0–9)	OS, DFS	8
Flørenes VA(b)[29]#	2015	Norway	81	Superficial spreading melanoma	NA	IHC	68.00	score >2 in cytoplasm and >0 in nucleus(range of 0–9)	OS	8
Chen KF[31]	2015	Taiwan	220	colorectal cancer	I-IV	IHC	41.40	H score ≥ 150(range of 0–300)	OS	7
Chen JS[12]	2015	China	37	advanced gastric cancer	>T2	IHC	≥50.00	appreciable staining >5% of target cells	OS	6
Tang QZ[35]	2015	China	131	ccRCC	I-IV	IHC	65.50	score ≥ 145(range of 0–300)	OS, DFS	6
Cristóbal[14]	2017	Spain	61	TNBC	I-III	IHC	72.10	H score ≥ 150(range of 0–300)	OS	8

IHC: Immunohistochemistry; RT-PCR: Real Time Polymerase Chain Reaction; NOS: Newcastle-Ottawa Scale; OS: overall survival; DFS: disease free survival; NSCLC: non-small-cell lung cancer; HCC: Hepatocellular Carcinoma; ccRCC: Clear Cell Renal Cell Carcinoma; NPC: Nasal Pharyngeal Cancer; TNBC: triple-negative breast cancer#; There were two parts of data (a and b) in each of the studies of Flørenes VA; NA: not available

### Evidence synthesis

**Fig 2:**
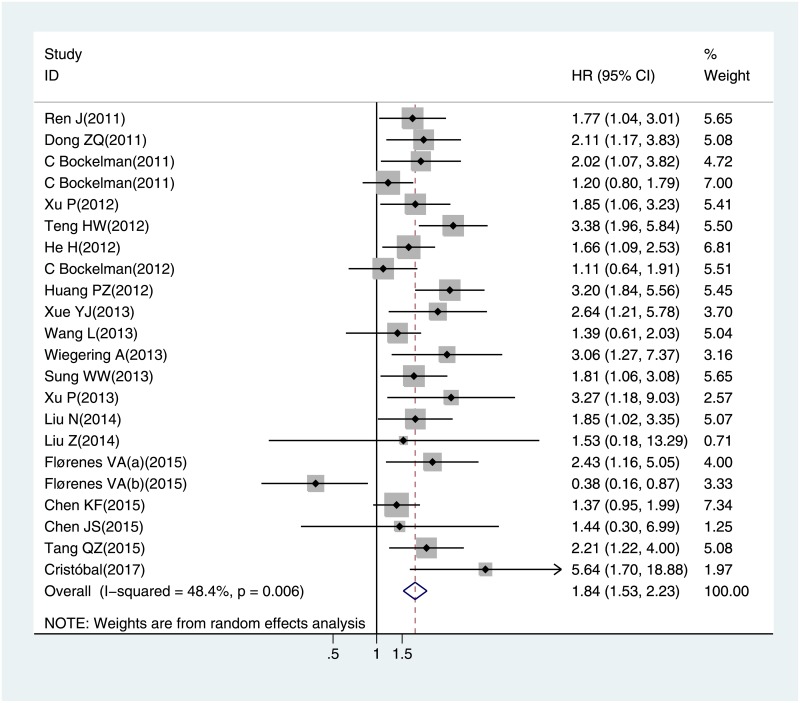
The correlation between CIP2A expression and overall survival (OS) in solid tumors.

**Fig 3:**
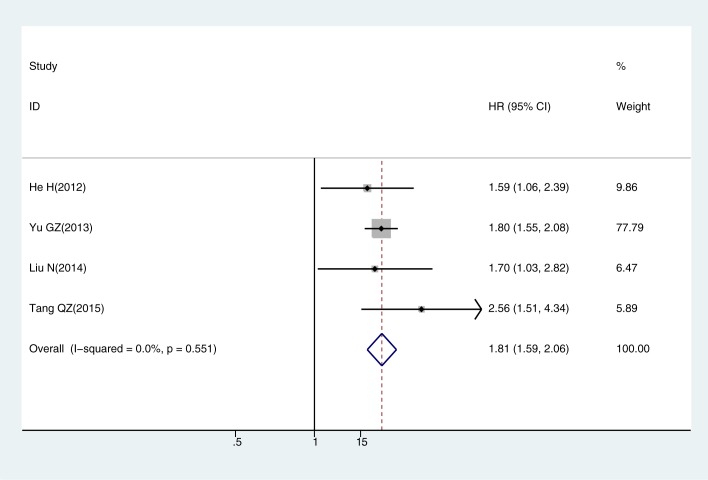
The correlation between CIP2A expression and disease free survival (DFS) in solid tumors.

The meta-analysis of CIP2A expression was based on two outcome endpoints: OS (overall survival) and DFS (Disease-free survival). The OS was defined as the time from diagnosis to death from any cause or to the date of the last follow-up, and the DFS defined as the time from randomization until the first event. For OS, twenty one studies were included in the meta-analysis. A random effect model was applied to calculate the pooled hazard ratio (HR) and 95% confidence interval (CI) on account of the heterogeneity test reported the P value of 0.006 and I^2^ statistic of 48.4%. The results shown that increased CIP2A expression was associated with poor OS in solid tumors (pooled HR = 1.844, 95% CI = 1.528–2.225, P<0.001) (Fig 2). Four studies included in the meta-analysis of DFS. The heterogeneity test reported the P value of 0.551 and I^2^ statistic of 0.0%, so the fixed-effects model was utilized. These results suggested a significant association between CIP2A expression and short DFS (pooled HR = 1.808, 95% CI = 1.591–2.055, P<0.001) (Fig 3). As the amount of datasets of DFS was all in China region, only the subgroup analysis within studies of OS was performed. The results suggested that the relationship between CIP2A over-expression and poor OS were significant in East-Asian populations (pooled HR = 1.92, 95% CI 1.85–2.24, P< 0.001) from 16 included studies, as well as in European group (pooled HR = 1.428, 95% CI 1.122–1.818, P = 0.004) from the other 6 studies. When stratified by cancer type, increased CIP2A expression had an unfavorable impact on the OS of patients with lung cancer (pooled HR = 1.842, 95% CI 1.286–2.638, P = 0.001) and colorectal cancer (pooled HR = 1.897, 95% CI 1.108–3.249, P = 0.020). Furthermore, we stratified the extracted data by the detection methods of CIP2A expression. There were 20 studies used IHC and two employed RT-PCR, the combined OS consistent presented an adverse prognostic effect of CIP2A expression (pooled HR = 1.781, 95% CI 1.433–2.212, P<0.001) in IHC group.

### Publication bias and sensitivity analysis

**Fig 4:**
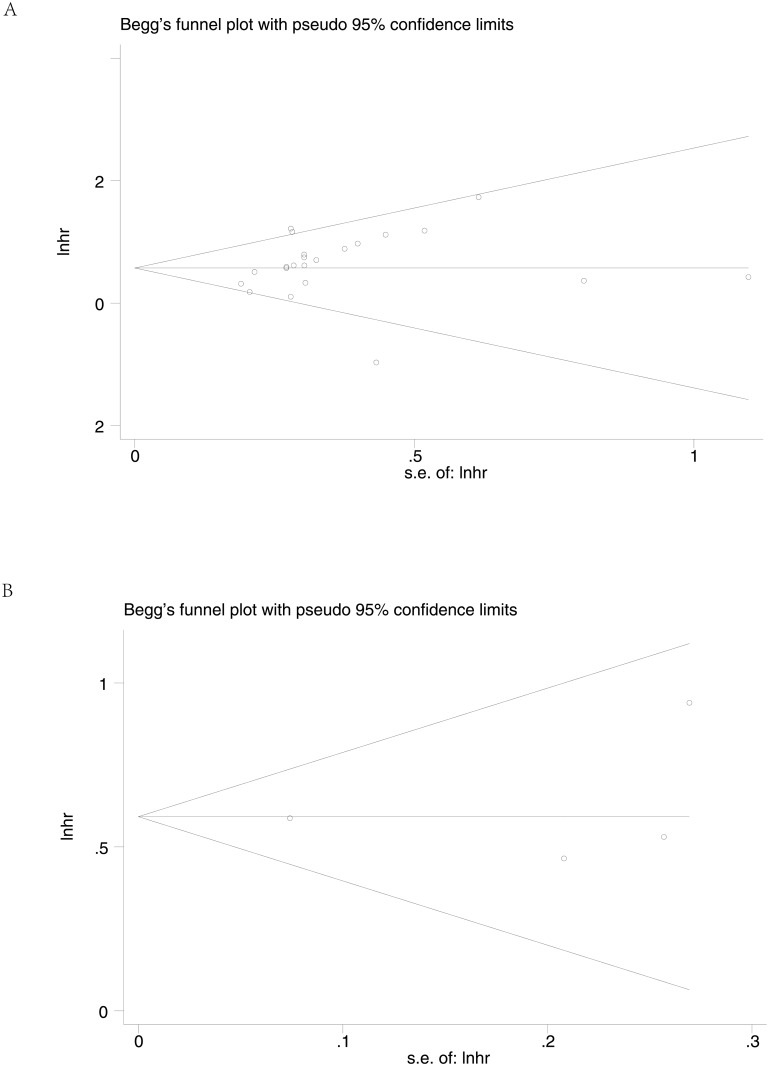
Begg’s funnel plots for the studies involved in the meta-analysis. (A) overall survival (B)disease free survival. Abbreviations: loghr, logarithm of hazard ratios; s.e., standard error.

**Fig 5:**
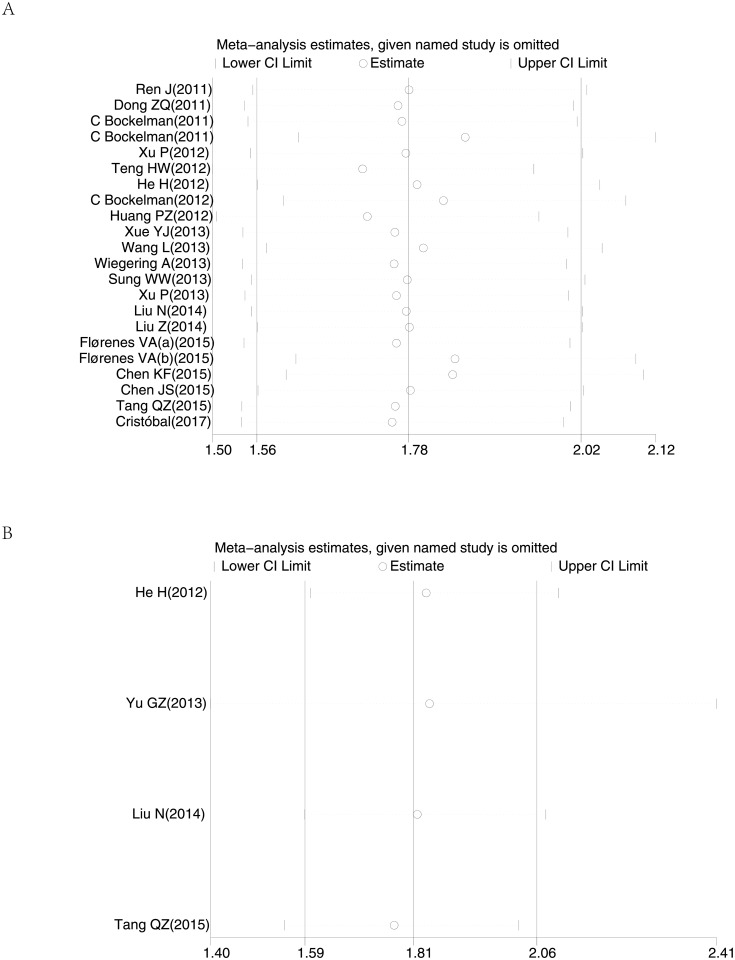
Sensitivity analysis of the meta-analysis. (A) overall survival (B) disease free survival.

Begg’s funnel plot and Egger’s test were used to estimate the publication bias in this meta-analysis. Visual inspection of the funnel plots revealed no evidence of obvious heterogeneity (Fig 4), and Egger’s tests regarding OS, DFS in the including studies (P = 0.247 and P = 0.761, respectively) showed non-significant values. Moreover, sensitivity analysis was carried out to assess the influence on the results described above. When excluding the dates of study in superficial spreading melanoma by the author Florenes VA et al.[29], the I^2^ statistic would reduce to 22.52% and the removal of any single study had no significant effect on the overall conclusion (Fig 5).

## Discussion

Increased CIP2A expression had been demonstrated at a high frequency in a number of solid tumors and the expression level could serve as a potential prognostic marker for OS and DFS. However, the association between the expression of CIP2A and its prognosis remains controversial. To our knowledge, this is the first meta-analysis to investigate the significance of CIP2A expression in the tumor patients.

Literature shown that CIP2A inhibited the tumor suppressor protein PP2A which downregulated phophorylation of AKT, a hallmark of cancers and stabilized the proto-oncogene, MYC in tumor cells [46]. CIP2A inhibits the activity of PP2A on MYC and enhance the MYC protein stability in tumor cell, which promotes the consequential proliferation and cancer progression. It has been known that MYC is one of the downstream target molecules of the PI3K-AKT-mTOR signaling pathway [47]. CIP2A enhances mTORC1 signaling pathway and leads to inhibition of PP2A and autophagy. CIP2A is also associated with the some stabilization phosphorylated PP2A substrates, including E2F1, DAPK1, Plk1 and AKT, resulting in the inhibition of senescence and apoptotic pathways [44]. Furthermore, PP2A acts as a negative regulator of MEK and ERK and PP2A inhibition by CIP2A is led to activation of these molecules. CIP2A interacts with Plk1, reduces the Plk1 ubiquitination and induces apoptosis [16]. Besides, CIP2A overexpression induces autoimmune response and promotes MKK4/7-JNK signaling pathway [17]. Based on the important role of CIP2A in regulating the numerous signaling pathways, it is easy to explain why CIP2A protein is associated with the prognosis of cancer, which may be served as a potential cancer therapy target.

There are several important implications in this meta-analysis based on our results. First, CIP2A over-expression was considered to be a poor prognostic marker in solid tumors. Thirteen different cancer types were included, containing lung cancer[21, 22, 30, 32], colorectal cancer[23, 27, 31, 37], renal cell carcinoma[20, 35], hepatocellular carcinoma[25, 34], gastric cancer[12], tongue cancer[24], bladder cancer[9], breast cancer[13, 14], nasal pharyngeal cancer[28], pancreatic ductal adenocarcinoma[26], melanoma[29], ovarian cancer[33] and cholangiocarcinoma[36]. The results showed that elevated CIP2A expression was significantly associated with poor OS and DFS in these performed types of cancer. Moreover, we demonstrated that different ethnic background has no obvious effect on the outcomes, as the similar prognostic significance of CIP2A2 over-expression in both East-Asian and European population. When we grouped the analysis, the HRs and 95%CIs for OS populations in East-Asia and Europe were different, indicating that the prognostic role of CIP2A for OS of solid tumors was more significant in East-Asian group, and CIP2A also had a stronger prognostic value. Finally, these findings indict the protein CIP2A could be a valuable therapeutic target, yet it needs to be further studied.

In spite of the strong results of our study, there are still some limitations in this meta-analysis should be carefully considered. First, although the study in superficial spreading melanoma by Florenes VA et al.[29] was identified as the main source of heterogeneity, the other specific clinical features contributed to the heterogeneity were not definite. Second, most of the studies were designed as retrospective and many studies preferred to publish the positive results. Third, only studies in English were enrolled in this meta-analysis. Forth, studies lacking sufficient data were also excluded. Last but one, the HR of some studies was not provided directly. We had to extract data from Kaplan-Meier curves and the result might have a little difference. Finally, the detection methods for assessing CIP2A expression and the cutoff values were inconsistent. Due to the method for assessing CIP2A positivity was not unified, when using the IHC method, the combined OS presented a poor prognostic effect of CIP2A expression. We were not able to evaluate the prognostic value of CIP2A by the RT-PCR method in the subgroup analysis due to a lack of data. Immunohistochemical staining of CIP2A was evaluated using a semiquantitative scoring system for both staining intensity and the percentage of positive cells. Staining intensity was graded as scored as “0” (no staining), “1” (weakly stained), “2” (moderately stained), and “3” (strongly stained). Most of the studies detected the CIP2A expression by IHC, the antibody concentrations and the cutoff values varied across different studies which might influence the results, and cause some biases in pooled analysis. Consequently, our estimation of the associations between overexpression of CIP2A and outcomes may have been overestimated.

In conclusion, based on the results of related scientific studies, our meta-analysis integrate the results of individual studies, which clearly reveal that high expression of CIP2A in solid tumor is significantly associated with poor prognosis. We propose the CIP2A should be regarded as a prognostic biomarker and a potential therapeutic target for solid tumors. Nevertheless, the clinical utility of CIP2A needs to be confirmed by well-designed and large sample size investigations in the future.

## Supporting information

S1 Checklist PRISMA 2009 of this paper. (PDF)
